# Mistargeting of secretory cargo in retromer-deficient cells

**DOI:** 10.1242/dmm.046417

**Published:** 2021-01-22

**Authors:** Sarah D. Neuman, Erica L. Terry, Jane E. Selegue, Amy T. Cavanagh, Arash Bashirullah

**Affiliations:** Division of Pharmaceutical Sciences, University of Wisconsin-Madison, Madison, WI 53705-2222, USA

**Keywords:** Retromer, Regulated exocytosis, Secretory granule, Salivary gland, *Drosophila*, *Vps26*

## Abstract

Intracellular trafficking is a basic and essential cellular function required for delivery of proteins to the appropriate subcellular destination; this process is especially demanding in professional secretory cells, which synthesize and secrete massive quantities of cargo proteins via regulated exocytosis. The *Drosophila* larval salivary glands are composed of professional secretory cells that synthesize and secrete mucin proteins at the onset of metamorphosis. Using the larval salivary glands as a model system, we have identified a role for the highly conserved retromer complex in trafficking of secretory granule membrane proteins. We demonstrate that retromer-dependent trafficking via endosomal tubules is induced at the onset of secretory granule biogenesis, and that recycling via endosomal tubules is required for delivery of essential secretory granule membrane proteins to nascent granules. Without retromer function, nascent granules do not contain the proper membrane proteins; as a result, cargo from these defective granules is mistargeted to Rab7-positive endosomes, where it progressively accumulates to generate dramatically enlarged endosomes. Retromer complex dysfunction is strongly associated with neurodegenerative diseases, including Alzheimer's disease, characterized by accumulation of amyloid β (Aβ). We show that ectopically expressed amyloid precursor protein (APP) undergoes regulated exocytosis in salivary glands and accumulates within enlarged endosomes in retromer-deficient cells. These results highlight recycling of secretory granule membrane proteins as a critical step during secretory granule maturation and provide new insights into our understanding of retromer complex function in secretory cells. These findings also suggest that missorting of secretory cargo, including APP, may contribute to the progressive nature of neurodegenerative disease.

## Abstract (precis)

**Summary:** Using *Drosophila* larval salivary glands as a model system, we show a new role for retromer in trafficking of secretory membrane and cargo proteins during regulated exocytosis, which could be relevant in neurodegenerative diseases.

## INTRODUCTION

A critical component of the regulated exocytosis machinery is the secretory granule; these specialized organelles contain cargo proteins surrounded by a phospholipid bilayer embedded with secretory granule membrane fusion proteins. Regulated exocytosis begins with the biogenesis of secretory granules at the *trans-*Golgi network (TGN). Nascent secretory granules initially bud from the TGN as immature granules; these immature granules then undergo a maturation process that renders them competent for exocytosis (Bonnemaison et al., 2013; Kögel and Gerdes, 2010). The specific processes that occur during maturation vary; however, several key steps occur in nearly all professional secretory cells, including homotypic fusion of immature granules to generate larger granules of a defined size (Bonnemaison et al., 2013; Tooze et al., 1991; Urbé et al., 1998; Wendler et al., 2001) and refinement of secretory granule cargo and membrane content (Bonnemaison et al., 2013; Kögel and Gerdes, 2010; Tooze and Tooze, 1986). Once maturation is completed, secretory granules are retained in the cytoplasm until an appropriate stimulus triggers their release. The release of secretory granule cargo is driven by membrane fusion proteins, such as SNAREs and Synaptotagmins, that are present on both the secretory granule membrane and the plasma membrane (Burgoyne and Morgan, 2003; Hong, 2005). These proteins catalyze fusion of the two disparate membranes to allow secretory granule cargo release.

The retromer complex is a highly conserved regulator of retrograde trafficking. This pathway is used to retrieve specific transmembrane proteins from endosomes and recycle them back to the TGN or the plasma membrane, thereby preventing their degradation in lysosomes (Burd and Cullen, 2014; Seaman et al., 1998; van Weering et al., 2010). Retromer is composed of two functional subcomplexes: the tubulation complex and the cargo-selective complex (CSC) (Small and Petsko, 2015; van Weering et al., 2010). The tubulation complex is composed of a heterodimer of two Bin/Ampiphysin/Rvs (BAR) domain-containing sorting nexins (Vps5 and Vps17 in yeast; SNX1/2 and SNX5/6 in mammalian cells); these proteins are capable of inducing membrane deformations to form endosomal tubules (Seaman et al., 1998; van Weering et al., 2010, 2012). The CSC, composed of a heterotrimer of VPS26 (also known as VPS26A), VPS29 and VPS35, recognizes specific transmembrane proteins and directs them into endosomal tubules (Bonifacino and Hurley, 2008). The CSC then recruits accessory proteins to drive scission of small retromer-studded vesicles containing the transmembrane cargo; these vesicles are transported back to the TGN (Cullen and Korswagen, 2012). The CSC can also work with a different sorting nexin, SNX3, to mediate retrograde trafficking of transmembrane proteins from endosomes in a tubule-independent manner (Burd and Cullen, 2014; Harterink et al., 2011; Zhang et al., 2011). CSC binding to endosomes requires interaction with both Rab5 (also known as RAB5A), an early endosome protein, and Rab7, a late endosome protein (Rojas et al., 2008). In this manner, the retromer complex retrieves specific transmembrane cargoes from endosomal compartments and recycles them back to the TGN via endosomal tubules. Work by us and others has suggested that secretory granule membrane fusion proteins may traffic through endosomal compartments (Azouz et al., 2014; Burgess et al., 2012; Grabner et al., 2006; Harrison-Lavoie et al., 2006; Ma et al., 2020; Neuman and Bashirullah, 2018), raising the intriguing possibility that these proteins undergo retromer-dependent retrieval and recycling.

The *Drosophila* larval salivary glands synthesize and secrete mucin-like ‘glue’ proteins via regulated exocytosis at the onset of metamorphosis (Beckendorf and Kafatos, 1976; Korge, 1977). Biogenesis of these mucin-containing secretory granules begins at the mid-third-instar larval transition, ∼24 h before the onset of metamorphosis (Beckendorf and Kafatos, 1976; Biyasheva et al., 2001). Nascent granules mature via homotypic fusion, reaching a terminal size of 5-8 µm in diameter (Neuman and Bashirullah, 2018; Niemeyer and Schwarz, 2000; Reynolds et al., 2019). Mature granules then undergo steroid hormone-dependent exocytosis beginning ∼4 h before the onset of metamorphosis (Biyasheva et al., 2001; Kang et al., 2017). After exocytosis, mucins are expelled out of the lumen of the salivary gland and onto the surface of the animal, allowing the prepupa to adhere to a surface during metamorphosis. Notably, the larval salivary glands complete just one single, developmentally regulated cycle of secretory granule biogenesis, maturation and exocytosis over a 24 h period. Thus, by using fluorescently tagged mucins coupled with markers for secretory granule membrane proteins and other organelles, we can observe the temporal sequence of intracellular trafficking events that contribute to secretory granule maturation and exocytosis in living cells.

Here, we use the *Drosophila* larval salivary glands to uncover a role for the retromer complex in regulated exocytosis. Our data demonstrate that there is a developmentally regulated increase in endosomal tubulogenesis that coincides with the onset of secretory granule biogenesis. Importantly, nascent secretory granules in *Vps26* mutant cells lack the appropriate membrane proteins, and our data suggest that these secretory granule membrane proteins undergo retromer-dependent trafficking via endosomal tubules. In the absence of appropriate membrane proteins, cargo proteins are mistargeted to the endolysosomal system and aberrantly accumulate within dramatically enlarged Rab7-positive endosomes. Our data also indicate that transmembrane cargo proteins, such as amyloid precursor protein (APP), which undergoes regulated exocytosis in salivary gland cells, are similarly missorted to the endolysosomal system. Overall, these results highlight a new role for the retromer complex in maturation of secretory granules during regulated exocytosis and provide new insights into the etiology of known intracellular trafficking phenotypes in retromer-deficient cells.

## RESULTS

### Trafficking via endosomal tubules is developmentally regulated at the onset of cargo biogenesis

**Fig. 1.**
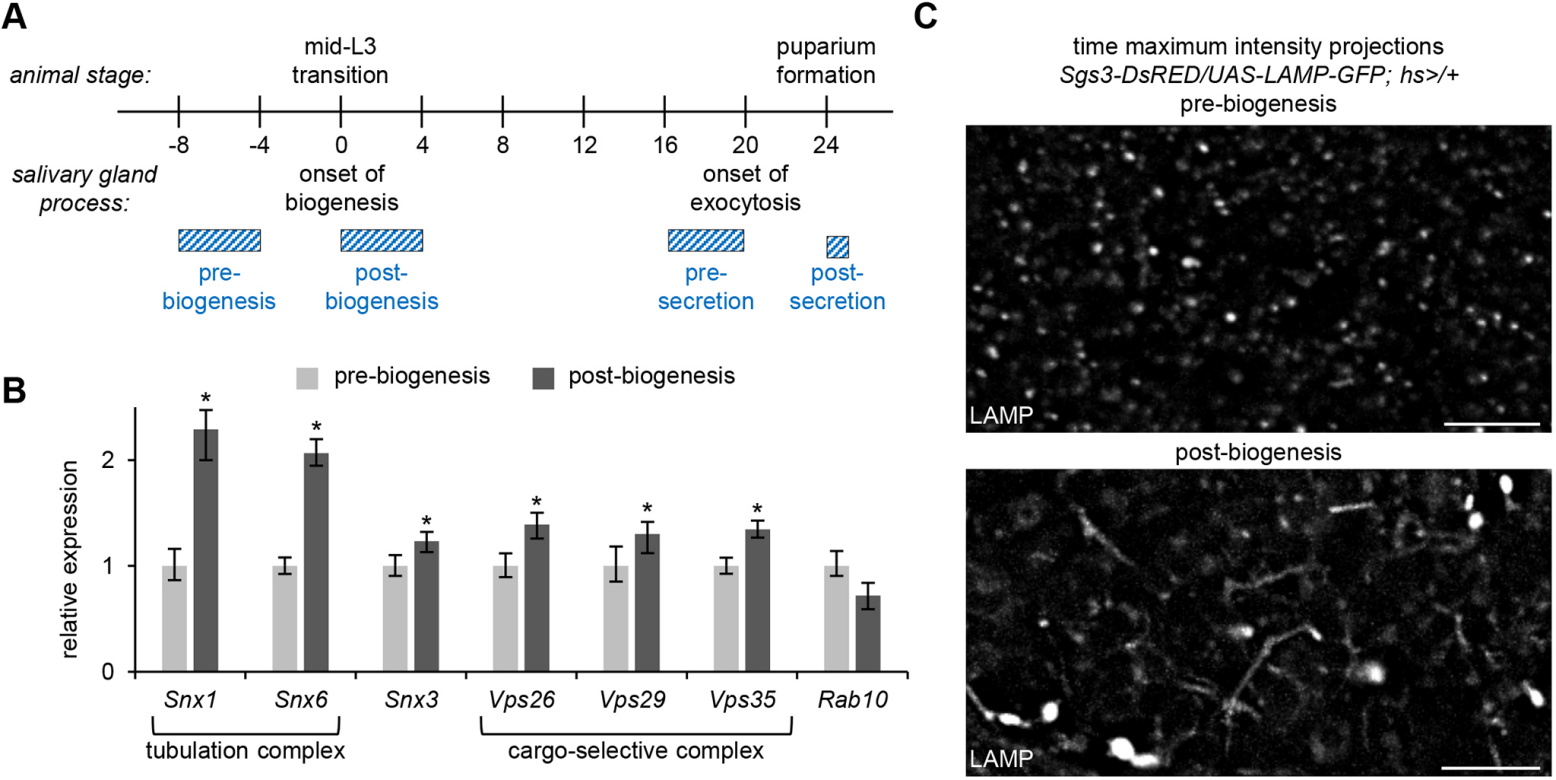
**Endosomal tubule activity increases at the onset of secretory granule biogenesis.** (A) Schematic depicting the timing of events during mucin-regulated exocytosis in the larval salivary glands. Biogenesis of mucin secretory granules begins at the mid-third instar (mid-L3) larval transition. Exocytosis begins ∼20 h later and is completed by puparium formation. Onset of biogenesis to completion of exocytosis spans 24 h. Developmental stages of salivary glands analyzed throughout this paper are depicted by the blue hatched bars. (B) qPCR analysis of retromer complex mRNA expression levels pre- and post-biogenesis of secretory granules shows significant upregulation of all retromer complex genes in post-biogenesis salivary glands, whereas *Rab10* expression levels do not significantly change. *y*-axis shows relative expression; *x*-axis shows the genes analyzed. Samples were run in biological triplicate and normalized to the reference gene *rp49*. Error bars and statistics were calculated by REST analysis; *P<0.05. (C) Live-cell time-lapse imaging of LAMP-GFP shows an increase in tubulogenesis in salivary glands post-biogenesis. Data shown as time maximum-intensity projections reflecting 250 timepoints (∼3 min total time) acquired from a single optical slice. Scale bars: 5 µm.

In the *Drosophila* larval salivary glands, biogenesis of mucins begins in the middle of the third larval instar, ∼20 h prior to their release via regulated exocytosis (Fig. 1A). To begin to test whether the retromer complex plays a role in this process, we used quantitative real-time PCR (qPCR) to measure mRNA expression levels of retromer complex components in dissected salivary glands isolated both before and after the onset of cargo biogenesis. The *Drosophila* genome contains a single ortholog of the CSC components *Vps26*, *Vps29* and *Vps35*, as well as three retromer-associated sorting nexins (*Snx1*, *Snx3* and *Snx6*) (Wang et al., 2014; Zhang et al., 2011). We observed a significant, ∼2-fold increase in the expression levels of *Snx1* and *Snx6* and smaller but still significant increases in the expression levels of *Snx3*, *Vps26*, *Vps29* and *Vps35* in glands after the onset of cargo biogenesis (Fig. 1B). By contrast, expression levels of *Rab10*, another endosome-localized protein (Chan et al., 2011; Gaudet et al., 2011), did not significantly change between pre- and post-biogenesis (Fig. 1B). Because *Snx1* and *Snx6* are BAR domain-containing proteins that are both necessary and sufficient for endosomal tubulogenesis (van Weering et al., 2012), this result may indicate that there is increased trafficking via endosomal tubules in salivary glands after the onset of cargo biogenesis. To test this, we used the endosome and lysosome marker LAMP-GFP (Burgess et al., 2012; Wang et al., 2014) for live-cell time-lapse imaging to assess tubule formation. LAMP-GFP primarily localized in puncta in pre-biogenesis glands; these puncta appeared stable and little tubule activity was observed (Fig. 1C; Movie 1). However, in post-biogenesis glands, we observed a significant increase in both the appearance and movement of endolysosomal tubules (Fig. 1C; Movie 2), indicating that there is a developmentally regulated increase in endolysosomal tubular trafficking at the onset of cargo biogenesis.

### Endosomal tubules traffic secretory granule membrane proteins to nascent granules

**Fig. 2.**
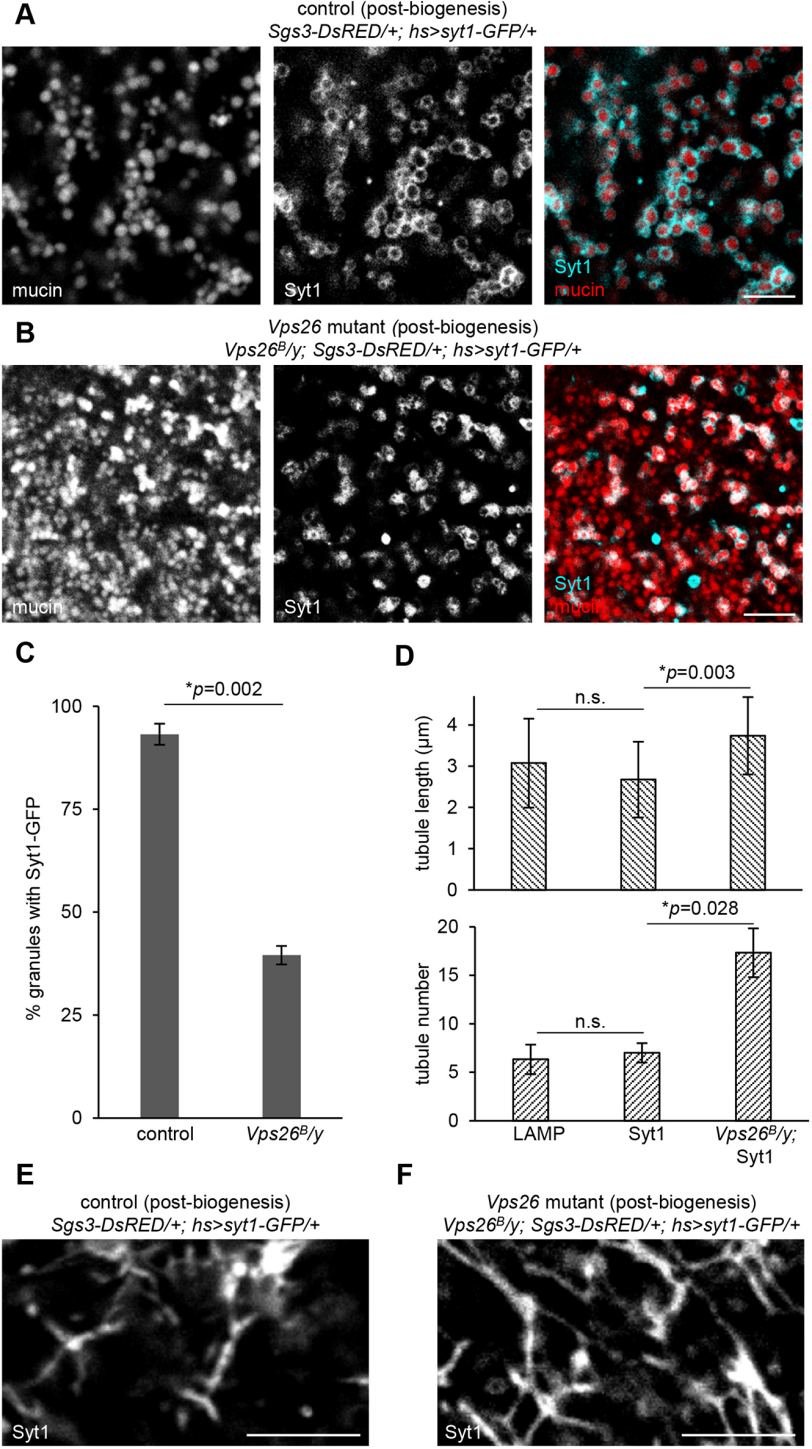
**Secretory granule membrane protein trafficking is disrupted in retromer mutant cells.** (A) Live-cell imaging of mucins (Sgs3-DsRED; red) and the secretory granule membrane protein Synaptotagmin-1-GFP (Syt1; cyan) in control salivary glands 0-4 h after the onset of secretory granule biogenesis shows that nascent granules are surrounded by Syt1. (B) Live-cell imaging of mucins and Syt1 in *Vps26* mutant cells shows that many mucin-containing vesicles lack Syt1 at biogenesis. (C) Quantification of the percentage of mucin-containing vesicles with Syt1-GFP in control and *Vps26* mutant cells. Graph shows mean±s.d. in three cells from three separate salivary glands. Mucin-containing vesicles counted: *n*=1282 in control and *n*=1566 in *Vps26* mutant. Statistics calculated by two-tailed paired Student's *t*-test. (D) Quantification of LAMP-GFP and Syt1-GFP tubule length and number in control cells and Syt1-GFP tubule length and number in *Vps26* mutant cells. Tubule length expressed as mean±s.d.; *n*=26 tubules per genotype. Tubule number expressed as mean±s.d. from three separate 100 µm^2^ areas. Statistics calculated by two-tailed paired Student's *t*-test. n.s., not significant. (E) Live-cell imaging of Syt1 in control cells shows that Syt1 localizes in a tubular network. Note that the salivary glands are composed of polarized epithelial cells, and tubules are localized near the basal membrane; few secretory granules are visible in this plane. (F) Live-cell imaging of Syt1 in *Vps26* mutant cells shows that Syt1 localizes in a more extensive tubular network in retromer-deficient cells. Scale bars: 5 µm.

Defects in retromer complex function are strongly associated with neurodegenerative diseases, including Alzheimer's disease and Parkinson's disease (Wang and Bellen, 2015). The genetic lesions associated with these diseases primarily map to CSC components (Muhammad et al., 2008; Small et al., 2005; Vilariño-Güell et al., 2011; Wen et al., 2011; Zimprich et al., 2011); therefore, we focused on analyzing CSC function to explore the role of the retromer complex in regulated exocytosis. We found that endogenous Vps35-TagRFP protein (Koles et al., 2016) partially co-localized with endogenous Rab7-EYFP (Dunst et al., 2015) in control glands (Fig. S1A), but Vps35-TagRFP was absent in the salivary glands of loss-of-function *Vps26* mutant animals (*Vps26^B^*; Wang et al., 2014) (Fig. S1B), indicating that the CSC is non-functional in this mutant background. To determine whether secretory granule biogenesis was affected by loss of retromer-dependent trafficking, we examined nascent secretory granules. Secretory granules are composed of two primary components: cargo proteins and membrane proteins. In the larval salivary glands, we can observe soluble secretory cargo proteins via fluorescent tagging of mucins (*Sgs3-DsRED*). We saw that mucins were produced in *Vps26* mutant cells, and the nascent granules appeared similar to those of controls at the onset of biogenesis (Fig. 2A,B). Similarly, we can observe secretory granule membrane proteins via fluorescent tagging of Synaptotagmin-1 (Syt1-GFP) (Neuman and Bashirullah, 2018). Nearly all nascent granules had Syt1-GFP on their membrane in control cells (Fig. 2A,C). By contrast, we found that more than half of the nascent granules in *Vps26* mutant cells did not have Syt1-GFP on their membrane (Fig. 2B,C), suggesting that there might be a defect in trafficking of secretory granule membrane proteins in retromer mutant cells.

Work by us and others has suggested that secretory granule membrane proteins traffic through the endosomal system (Azouz et al., 2014; Burgess et al., 2012; Grabner et al., 2006; Harrison-Lavoie et al., 2006; Ma et al., 2020; Neuman and Bashirullah, 2018). We therefore wanted to test whether Syt1-GFP trafficked through endosomal tubules. Strikingly, we saw that Syt1-GFP localized in a tubular network that closely resembled that of LAMP-GFP in post-biogenesis control cells (Fig. 2D,E). We also observed an expansion of this Syt1-GFP tubular network in *Vps26* mutant cells (Fig. 2D,F). Because SNX1 protein expression has been reported to increase in mammalian VPS26 mutant cells (Seaman, 2004), we would expect to see more endosomal tubules in the absence of CSC function. Taken together, these results suggest that secretory granule membrane proteins undergo retromer-dependent trafficking through the endolysosomal network, and that this trafficking is required for delivery of membrane proteins to nascent secretory granules.

### Nascent granules lacking appropriate membrane proteins become enlarged and are not secreted

**Fig. 3.**
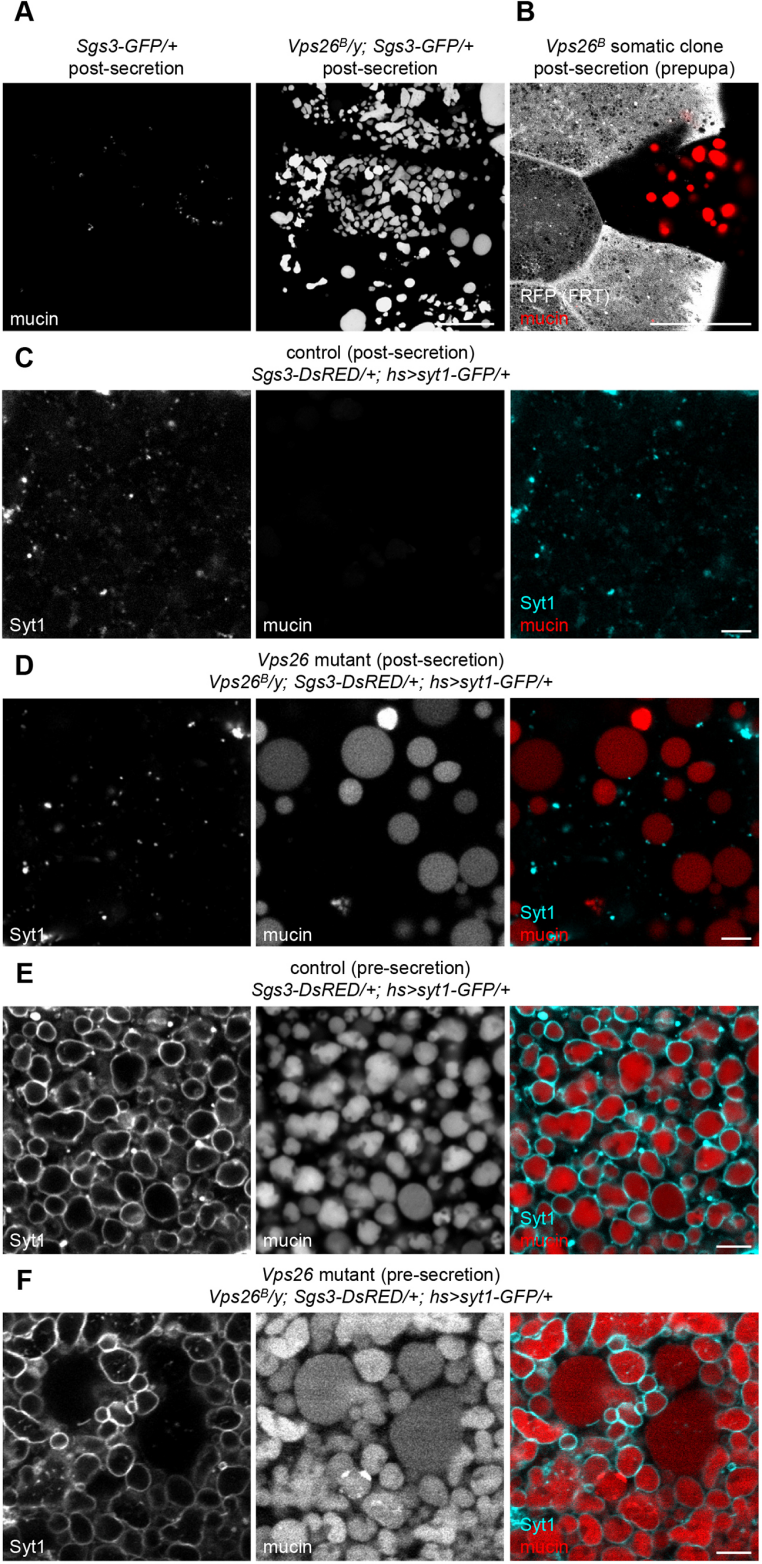
**Regulated exocytosis of secretory cargo is disrupted in retromer mutant cells.** (A) Live-cell imaging of mucins (Sgs3-GFP) in control and *Vps26* mutant salivary glands post-secretion. All mucin cargo proteins are secreted in control cells, whereas *Vps26* mutant cells still contain large amounts of cargo. (B) *Vps26^B^* somatic clone shows that mucin secretion defects are cell autonomous. The mutant clone is marked by loss of RFP, shown in gray, and mucins are shown in red. Note that the mutant clone is the only cell still containing mucins in prepupal salivary glands. Full genotype: *Vps26^B^,* *FRT19A/hs-flp, UbiRFPnls, FRT19A; Sgs3-GFP/+*. (C) Live-cell imaging of mucins (Sgs3-DsRED; red) and Syt1-GFP (cyan) in control salivary gland cells post-secretion. Mucins have been secreted, and Syt1 localizes in small puncta within the cytoplasm. (D) Live-cell imaging of mucins and Syt1 in *Vps26* mutant cells shows that a significant number of mucin-containing vesicles are not secreted; these vesicles are also enlarged and lack Syt1 on their membrane. (E) Live-cell imaging of mucins and Syt1 in control cells pre-secretion. All mucin-containing secretory granules have Syt1 on their membrane, and granules are of a relatively uniform size. (F) Live-cell imaging of mucins and Syt1 in *Vps26* mutant cells pre-secretion. Many normal-sized mucin-containing secretory granules have Syt1 on their membrane; however, enlarged mucin-containing vesicles lack Syt1. Scale bars: 50 µm (A,B); 5 µm (C-F).

Secretory granule membrane proteins, including Synaptotagmins and SNAREs, are essential for fusion with the plasma membrane during regulated exocytosis. Given that many nascent granules lacked Syt1-GFP at biogenesis in *Vps26* mutant cells, we tested whether there were any regulated exocytosis defects in these cells. Live-cell imaging of mucins at puparium formation (PF), the developmental stage when all mucins should be secreted, revealed that a great deal of cargo was retained in the salivary gland cells of *Vps26^B^* mutant animals (Fig. 3A). *Vps26* mutant *flp/FRT* somatic clones in the salivary glands also displayed retention of mucins at prepupal stages (Fig. 3B), indicating that a considerable amount of mucins are never secreted and that these defects are cell autonomous.

Importantly, imaging of *Vps26* mutant salivary glands at PF (post-secretion) demonstrated that retained mucin-containing vesicles lacked Syt1-GFP on their membranes (Fig. 3C,D). These vesicles also appeared to be significantly enlarged compared to the maximum size of mucin granules in control cells pre-secretion (compare Fig. 3D versus E). We also analyzed the morphology and membrane protein content of granules immediately prior to secretion, when maturation is complete and granules have reached their final size. At this stage in control cells, all cargo-containing secretory granules had Syt1-GFP on their membrane and were of a relatively uniform size (Fig. 3E); however, in *Vps26* mutant cells, we observed a population of enlarged, mucin-containing vesicles that did not have Syt1-GFP on their membrane, although the remaining granules of normal size did have Syt1-GFP (Fig. 3F). Therefore, our results thus far demonstrate that two distinct populations of mucin-containing vesicles develop in retromer-deficient cells. First is a population of secretory granules that have appropriate membrane proteins at biogenesis; these achieve normal size and are secreted. Second is a population of vesicles that lack the appropriate membrane proteins at biogenesis; here, mucins are present within enlarged vesicles that are not secreted.

### Secretory cargo accumulates in enlarged Rab7-positive endosomes

**Fig. 4.**
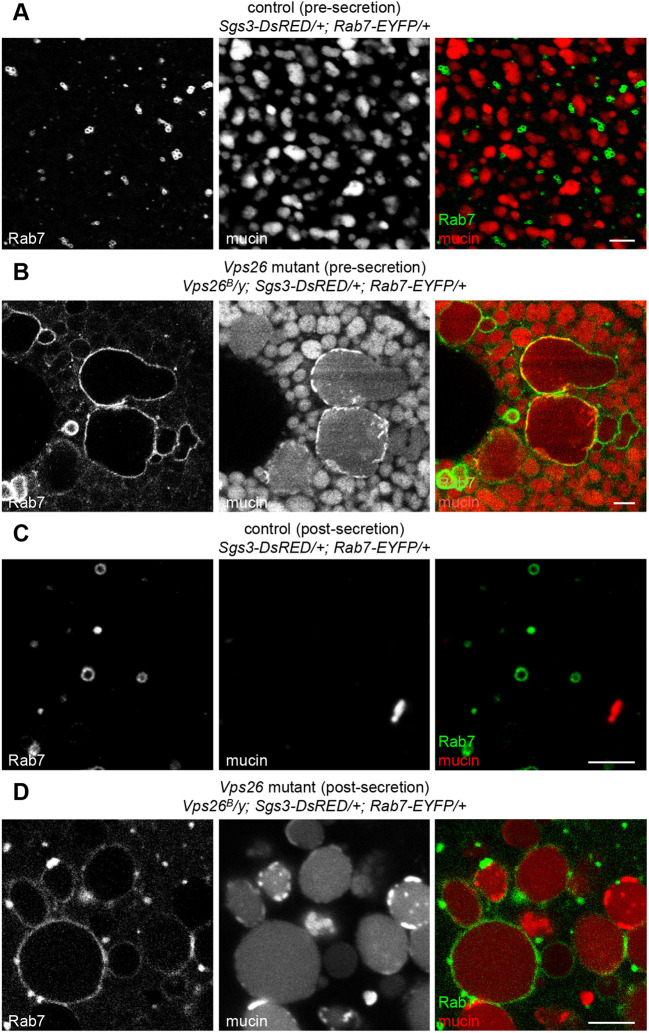
**Enlarged Rab7-positive endosomes contain secretory cargo proteins in retromer mutant cells.** (A) Live-cell imaging of Rab7-EYFP (green) and mucins (Sgs3-DsRED; red) in control salivary glands pre-secretion. Rab7 localizes in small puncta that are completely distinct from mucins. (B) Live-cell imaging of Rab7 and mucins in *Vps26* mutant salivary glands pre-secretion. Note that Rab7-positive endosomes are dramatically enlarged and contain mucin cargo proteins. (C) Live-cell imaging of Rab7 and mucins in control salivary glands post-secretion. Most mucins have been secreted and Rab7 localizes in small vesicles. (D) Live-cell imaging of Rab7 and mucins in *Vps26* mutant salivary glands post-secretion. Unsecreted mucin-containing vesicles are dramatically enlarged and surrounded by Rab7. Scale bars: 5 µm.

Because homotypic fusion during maturation requires secretory granule membrane proteins, nascent granules lacking Syt1-GFP in *Vps26* mutant cells should not be able to undergo this process. Therefore, the dramatically enlarged vesicles observed just prior to and after secretion must reach this size by other mechanisms. Because the retromer complex is known to regulate retrograde trafficking from Rab5/Rab7-positive endosomes (Rojas et al., 2008), we first analyzed the localization and morphology of these endosomes using endogenously-regulated, EYFP-tagged Rab5 and Rab7 (Dunst et al., 2015). There were no significant differences in the localization or morphology of Rab5-positive early endosomes in *Vps26* mutant cells (Fig. S2A); however, Rab7-positive late endosomes were dramatically enlarged and often misshapen in salivary gland cells from *Vps26* mutant animals and in *Vps26* mutant *flp/FRT* somatic clones (Fig. S2B,C). Strikingly, these enlarged, Rab7-positive endosomes contained mucins in both pre- and post-secretion *Vps26* mutant salivary gland cells (Fig. 4B,D), whereas mucins were not seen inside Rab7-positive endosomes in control cells (Fig. 4A,C). To confirm these results, we also assessed the localization of mucins with a GFP-tagged FYVE domain (FYVE-GFP), which binds to the lipid phosphatidylinositol-3-phosphate (PI3P) present in endosomal membranes (Wucherpfennig et al., 2003). Consistent with our Rab7 results, we observed enlarged, mucin-containing vesicles wrapped in FYVE-GFP in *Vps26* mutant glands both pre- and post-secretion (Fig. S3). Taken together, these results suggest that soluble cargo proteins accumulate in the endosomal system in retromer mutant cells.

### Accumulation of secretory cargo proteins within endosomes begins immediately following secretory granule biogenesis

**Fig. 5.**
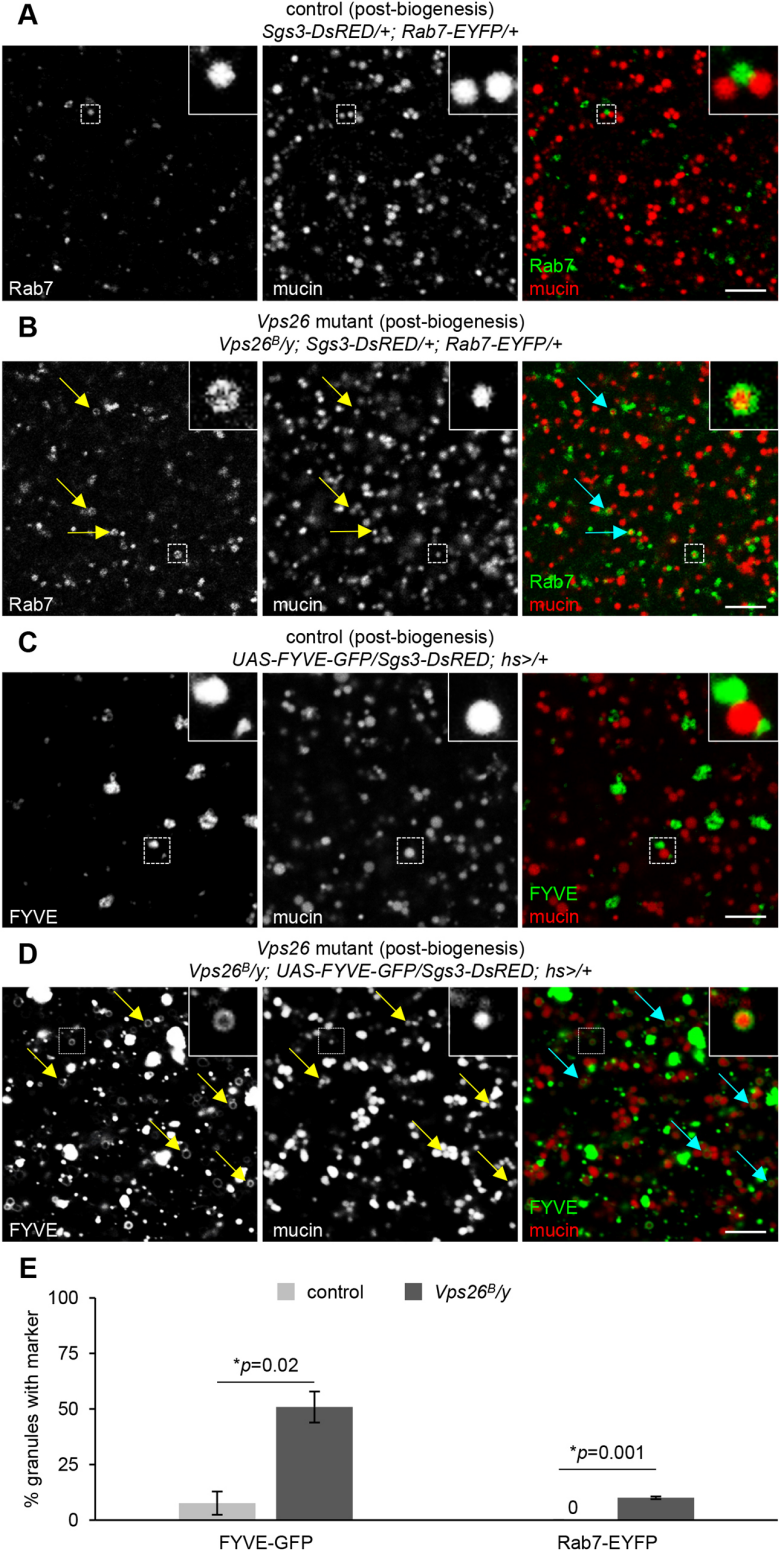
**Secretory cargo begins to accumulate within endosomes at biogenesis.** (A) Live-cell imaging of Rab7-EYFP (green) and mucins (Sgs3-DsRED; red) in control cells immediately following the onset of secretory granule biogenesis. Rab7 localizes in small puncta that are completely distinct from mucins. Boxed areas are magnified at the upper right of images. (B) Live-cell imaging of Rab7 and mucins in *Vps26* mutant cells immediately following the onset of secretory granule biogenesis. Rab7 endosomes appear slightly enlarged compared to controls (A) and some contain mucin cargo, indicated by arrows. Boxed areas are magnified at the upper right of images. (C) Live-cell imaging of the PI3P marker FYVE-GFP (green) and mucins (Sgs3-DsRED; red) in control salivary glands post-biogenesis. FYVE-GFP and mucins are completely distinct; boxed areas are magnified at the upper right of images. (D) Live-cell imaging of FYVE-GFP and mucins in *Vps26* mutant salivary glands post-biogenesis. A significant number of mucin-containing vesicles are surrounded by FYVE-GFP, indicated by arrows. Boxed areas are magnified at the upper right of images. Scale bars: 5 µm. (E) Quantification of the percentage of mucin-containing vesicles surrounded by FYVE-GFP or Rab7-EFYP shows a significant increase in *Vps26* mutant cells. Note that no mucin-containing vesicles were surrounded by Rab7-EYFP in control cells. Graphs show mean±s.d. of the percentage of mucin-containing vesicles surrounded by FYVE-GFP or Rab7-EYFP in cropped images of three cells from three separate salivary glands for each genotype. Mucin-containing vesicles counted: FYVE-GFP, control *n*=93; FYVE-GFP, *Vps26* mutant *n*=120; Rab7-EYFP, control *n*=91; Rab7-EYFP, *Vps26* mutant *n*=79. Statistics calculated by two-tailed paired Student's *t*-test.

Our next goal was to determine when endosomal accumulation of mucins begins in *Vps26* mutant cells. The larval salivary glands enable us to answer this question because we can observe the entire process of regulated exocytosis unfold sequentially in real time. Because we observed Syt1-GFP trafficking defects at secretory granule biogenesis, we tested whether mucin trafficking was also affected at this stage. In control cells, Rab7 localized in small puncta that were completely distinct from nascent granules during secretory granule biogenesis (Fig. 5A,E). In contrast, *Vps26* mutant cells exhibited about a 10-fold increase in the percentage of enlarged, Rab7-positive vesicles containing mucins at this same developmental stage (Fig. 5B,E). To confirm these results, we also analyzed the localization of FYVE-GFP with mucin cargo proteins. Like Rab7, FYVE-GFP localized in puncta that were largely distinct from nascent secretory granules in control cells (Fig. 5C,E), while *Vps26* mutant cells had a significant increase in mucins surrounded by FYVE-GFP (Fig. 5D,E). Interestingly, we observed a temporal separation in the trafficking phenotypes in retromer-deficient cells. Mucin-containing vesicles lacking Syt1-GFP were visible immediately at the onset of secretory granule biogenesis, whereas mucin-containing FYVE-GFP- and Rab7-EYFP-positive endosomes did not appear until about 4 h after the onset of biogenesis, suggesting that mucin cargo proteins lacking Syt1-GFP are taken up by endosomes after nascent granules have budded from the TGN. These results indicate that soluble mucin cargo proteins lacking appropriate membrane proteins may be aberrantly missorted to the endolysosomal system in retromer-deficient cells.

The retromer complex recycles the cation-independent mannose-6 phosphate receptor (CI-M6PR; also known as IGF2R), a carrier protein for lysosomal hydrolases (Arighi et al., 2004; Seaman, 2004). Without recycling of CI-M6PR, lysosomal hydrolases are not efficiently delivered, resulting in lysosomal dysfunction (McMillan et al., 2017). To test whether lysosomal dysfunction results in accumulation of mucin cargo proteins within endosomes, we looked for retention of mucins in loss-of-function *Lerp* (the fly ortholog of *CI-M6PR*) mutant cells (Hasanagic et al., 2015). Importantly, *Lerp* mutant salivary glands did not contain any mucin cargo proteins at the onset of metamorphosis (Fig. S4B), indicating that there are no secretion defects in this mutant background. To confirm that *Lerp* mutant salivary glands did have lysosomal dysfunction, we used LysoTracker staining to label acidified organelles. Control glands at PF contained many small LysoTracker-positive puncta, consistent with the expected size of acidified late endosomes and lysosomes (Fig. S4A). By contrast, *Lerp* mutant salivary glands contained enlarged LysoTracker-positive organelles that appeared dense in a differential interference contrast (DIC) image (Fig. S4B). These phenotypes confirm that there is lysosomal dysfunction in *Lerp* mutant glands. Overall, these data suggest that lysosomal dysfunction is not the cause of aberrant mucin cargo protein accumulation in retromer mutant cells; instead, they suggest that mucins are mistargeted to the endosomal system upon loss of retromer complex function.

### Membrane-bound cargo proteins, like APP, are also mistargeted to the endolysosomal system in retromer mutant cells

**Fig. 6.**
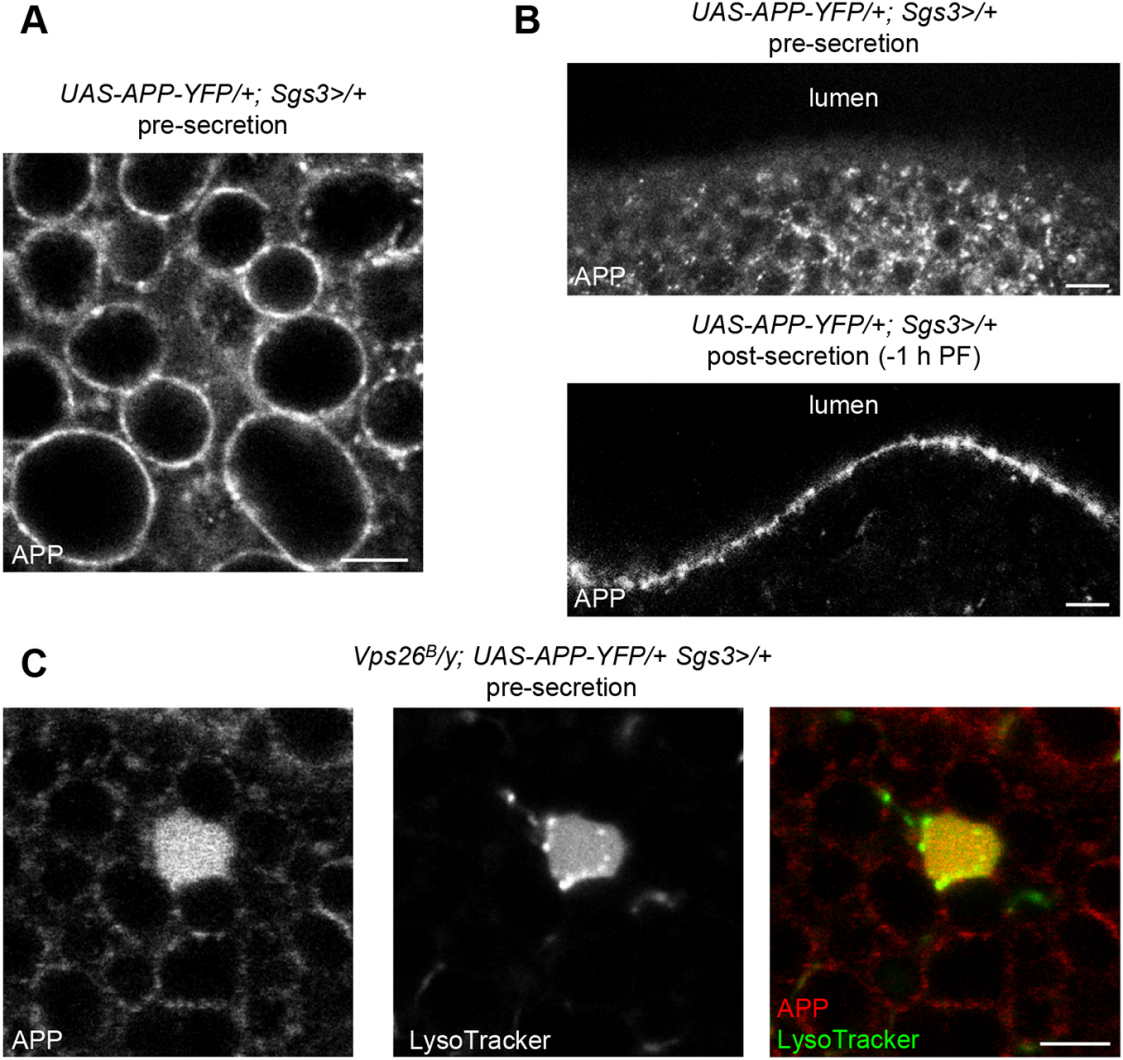
**APP undergoes regulated exocytosis and is missorted to endosomes in retromer mutant cells.** (A) Live-cell imaging of APP-YFP in control salivary glands pre-secretion. Note that APP is enriched on mucin secretory granule membranes. (B) Live-cell imaging of APP localization in control glands pre- and post-secretion. The apical membrane of the salivary glands faces the lumen (top of images). APP is absent from the apical membrane pre-secretion but is enriched on the apical membrane post-secretion, indicating that APP undergoes regulated exocytosis in salivary gland cells. (C) Live-cell imaging of APP (red) and LysoTracker (green) to label acidified organelles in *Vps26* mutant cells. APP accumulates in enlarged, LysoTracker-positive compartments in *Vps26* mutant cells pre-secretion. Note that image exposure was adjusted to avoid significant oversaturation of the enlarged compartment containing APP-YFP. Each image shows a portion of a single salivary gland cell. Scale bars: 5 µm.

Defects in retromer complex function are strongly associated with neurodegenerative diseases, including Alzheimer's disease and Parkinson's disease (Muhammad et al., 2008; Small et al., 2005; Wang and Bellen, 2015; Wen et al., 2011). One of the hallmarks of Alzheimer's disease is the accumulation of amyloid-β (Aβ) plaques (Stelzmann et al., 1995); Aβ is a cleavage product of endosome-associated processing of APP (Reiss et al., 2018), a membrane-bound, secreted protein that is known to undergo retromer-dependent retrograde trafficking via recycling of the Sortilin-LA (SorLA; also known as SORL1) sorting receptor (Fjorback et al., 2012; Lane et al., 2012; Wang and Bellen, 2015). In Alzheimer's disease, it is thought that retromer loss of function results in failure to recycle SorLA, resulting in retention of APP in endosomes, where APP can be processed by β- and γ-secretase to generate aberrantly large quantities of Aβ (Small and Gandy, 2006). However, our data suggest that there may be additional mechanisms that lead to endosomal accumulation of APP. First, we found that ectopically expressed human APP is secreted via regulated exocytosis in salivary gland cells. Regulated exocytosis of mucin granules occurs at the apical membrane of the glands (Biyasheva et al., 2001; Rousso et al., 2015; Tran et al., 2015), and we observed that APP was absent from the apical membrane but present on mucin granule membranes in control cells prior to the onset of secretion (Fig. 6A,B). However, after the onset of mucin secretion, APP was present on the apical membrane (Fig. 6B), indicating that it had been deposited there during regulated exocytosis. We have previously shown that other plasma membrane-localized proteins, including mCD8-GFP, do not accumulate on secretory granule membranes when overexpressed (Kang et al., 2017), indicating that this phenotype is specific to APP. Additionally, endogenous APP localizes to synaptic vesicles and is secreted via regulated exocytosis in neurons (Groemer et al., 2011), highlighting an important parallel between our salivary gland system and neuronal cells. We then examined the localization of APP in *Vps26* mutant cells. Pre-secretion, we found that APP was still present on secretory granule membranes; however, it was also present in large clumps that co-localized with LysoTracker staining (Fig. 6C), indicating that these are likely enlarged, acidified late endosomes or lysosomes. These results demonstrate that ectopically expressed APP is secreted to the plasma membrane via regulated exocytosis in control salivary gland cells; however, in retromer mutant cells, APP accumulates in the endolysosomal system prior to its appearance on the plasma membrane. These data suggest that APP is directly trafficked to the endolysosomal system in retromer-deficient cells. Taken together, these results suggest that both soluble and membrane-bound secretory cargo proteins are mistargeted to endosomes during regulated exocytosis in retromer mutant cells.

## DISCUSSION

Professional secretory cells synthesize massive quantities of cargo proteins that are destined for regulated exocytosis. Therefore, it is imperative that these cells exercise careful control over the delivery of proteins to the appropriate subcellular locations. Here, we have identified a role for the retromer complex in recycling of secretory granule membrane proteins to nascent granules; delivery of these membrane proteins promotes secretory granule maturation and competence for secretion. Failure to deliver secretory granule membrane proteins to nascent granules results in the mistargeting and progressive accumulation of secretory cargo proteins within endosomes. These results reveal new insights into our understanding of retromer loss-of-function phenotypes and their role in disease etiology.

One of the critical steps of secretory granule maturation in many different cell types is the homotypic fusion of immature granules to generate larger, mature granules of a defined size (Bonnemaison et al., 2013; Kögel and Gerdes, 2010). These membrane fusion events are mediated by secretory granule membrane proteins, like Synaptotagmins and SNAREs; therefore, the presence of these proteins on nascent granules is obligatory for maturation to proceed. Synaptotagmins and SNAREs are also required later for secretory granule fusion with the plasma membrane during regulated exocytosis. Currently, it is thought that both secretory granule membrane proteins and secretory granule cargo proteins are concurrently produced via *de novo* synthesis; these proteins are translated in the rough endoplasmic reticulum (ER), trafficked through the Golgi cisternae, and packaged into nascent granules via budding from the TGN. Our results partially support this model, as we observe that some nascent secretory granules do possess the appropriate membrane proteins in retromer-deficient cells; these granules appear to mature normally and are eventually secreted. However, we also observe that many nascent granules lack the proper membrane proteins in retromer mutant cells, suggesting that there is an alternative, retromer-dependent pathway for delivery of secretory granule membrane proteins. In this alternative pathway, secretory granule membrane proteins are recycled through endosomal compartments to nascent granules. Our data suggest that this retromer-dependent recycling pathway is critical during regulated exocytosis, as a significant fraction of nascent secretory granules rely on it to acquire the proper membrane proteins during maturation. Previous work has shown that retromer complex function is required to deliver membrane-bound melanogenic enzymes to melanosomes (McGough et al., 2014), suggesting that the retromer complex plays a functional role in regulated exocytosis in many different cell types.

Although our data suggest that secretory granule membrane proteins are new cargo proteins for the retromer complex, the mechanism by which retromer recognizes these proteins remains unclear. Typically, the Vps26-Vps29-Vps35 heterotrimer that composes the cargo-selective complex, is, as the name implies, responsible for identification of transmembrane cargo proteins that undergo retrograde trafficking/recycling. Identification and capture of cargo has typically been reported to require interaction between VPS35 and specific motifs on the cytoplasmic tail of transmembrane proteins (Arighi et al., 2004; Fjorback et al., 2012; Mukadam and Seaman, 2015; Nothwehr et al., 1999; Seaman, 2004, 2007). However, no specific ‘consensus’ sorting motif for CSC binding has been identified. Our data suggest that Synaptotagmin-1, and possibly other secretory granule membrane proteins, are new retromer cargo proteins. Future studies will be required to identify the mechanism by which the retromer complex identifies secretory granule membrane proteins, including analysis of potential sorting motifs.

Retromer complex dysfunction has frequently been reported to result in enlarged endosomes (Arighi et al., 2004; Maruzs et al., 2015; Wang et al., 2014). This defect has largely been attributed to failure to remove retromer cargo proteins from endosomes or deemed a passive consequence of impaired lysosomal function resulting from reduced delivery of lysosomal hydrolases. However, our results suggest an alternative explanation. We observe that secretory cargo proteins progressively accumulate within endosomal compartments, resulting in endosomes of enormous size. Our data suggest that this accumulation of cargo results from an active process where aberrant secretory cargo proteins lacking the appropriate membrane proteins are mistargeted to the endolysosomal system. Additionally, we do not observe accumulation of secretory cargo proteins when lysosomal function is disrupted by loss of the lysosomal hydrolase carrier. Therefore, we propose that mistargeting of secretory cargo is a novel etiological factor in the development of enlarged endosomes in retromer-deficient cells. Given that endosomal dysfunction is also strongly associated with neurodegenerative disease (Cataldo et al., 2000), our results could also provide new insights into the etiology and progressive nature of neurodegenerative disease.

## MATERIALS AND METHODS

### *Drosophila* stocks, husbandry, somatic clones and developmental staging

The following fly stocks were obtained from the Bloomington *Drosophila* Stock Center: *w^1118^*, *Sgs3-GFP*, *Rab5-EYFP*, *Rab7-EYFP*, *Sgs3-GAL4*, *hs-GAL4*, *UAS-GFP-LAMP*, *UAS-GFP-myc-2xFYVE*, *UAS-APP-YFP*, *UAS-Synaptotagmin-1-GFP*, *Vps26^B^*, *Vps35-TagRFP*, *hs-flp*, *UbiRFPnls*, *FRT19A* and *Lerp^F6^*. *Sgs3-DsRED* was provided by A. Andres (University of Nevada, Las Vegas, NV, USA). The full genotypes of animals analyzed in each figure can be found in Table S2. Note that animals from experiments using *hs-GAL4* were not heat-shocked; the *hs-GAL4* transgenic construct ‘leaks’ constitutively in the salivary glands owing to a cryptic promoter in the transgene (Costantino et al., 2008). All experimental crosses were grown on standard cornmeal-molasses media in uncrowded vials or bottles and maintained at 25°C. For *Vps26^B^* somatic clones in the salivary glands, embryos were heat-shocked at 37°C for 30 min 0-4 h after egg lay. Staged animals collected pre-secretion were selected as wandering L3 (wL3) larvae at about −8 h PF (∼4 h before the onset of secretion) and imaged under a fluorescent dissecting microscope to ensure that no glue protein was present in the lumen. Animals collected post-secretion were staged as white prepupae (0 h PF) for the onset of PF. For analyses pre- and post-mucin biogenesis, animals were collected 0-4 h after hatching from the embryo (0-4 h after L1) and allowed to age at 25°C for the appropriate time. Animals pre-biogenesis were collected at 56-60 h after L1 (8 h prior to the induction of glue biosynthesis). For 0-4 h after biogenesis, early L3 (eL3) larvae were collected and analyzed under a fluorescent dissecting microscope to confirm the absence of *Sgs3-GFP* or *Sgs3-DsRED* expression. Animals lacking mucin expression were then allowed to age for 4 h at 25°C, then were again analyzed for *Sgs3-GFP* or *Sgs3-DsRED* expression. Animals expressing the transgene at this time were collected for analysis.

### qPCR

qPCR was performed as previously described (Ihry et al., 2012). Total RNA was isolated from salivary glands at the appropriate developmental stage (see staging details in section above) using the RNeasy Plus Mini Kit (Qiagen). cDNA was synthesized from 400 ng total RNA using the SuperScript III First Strand Synthesis System (Invitrogen). Samples were collected and analyzed in biological triplicate, and minus reverse transcriptase controls were conducted to confirm the absence of genomic DNA contamination. qPCR was performed using a Roche LightCycler 480 with LightCycler 480 SYBR Green I Master Mix (Roche). Amplification efficiencies were calculated for each primer pair and minus template controls were included for each experiment. Relative Expression Software Tool (REST) was used to calculate relative expression and *P*-values (Pfaffl et al., 2002). Expression was normalized to the reference gene *rp49* (also known as *RpL32*); these primers were previously published (Ihry et al., 2012). Retromer complex and *Rab10* primers were designed using FlyPrimerBank (Hu et al., 2013); primer sequences are listed in Table S1.

### Confocal microscopy

All images were obtained from live, unfixed salivary glands; at least ten glands were imaged per experiment. True experimental blinding was not possible given the nature of the experiments and the genotypes analyzed; however, when possible, results were independently verified by another laboratory member who was blinded to the genotype of the sample. Imaging was carried out at room temperature. Salivary glands were dissected in PBS from animals of the appropriate genotype and developmental stage and mounted in either PBS or 1% low-melt agarose (Apex Chemicals); glands were imaged for no longer than 10 min after mounting. Images were acquired using either an Olympus FV1000 laser scanning microscope (40× oil immersion objective, NA 1.30; 60× oil immersion objective, NA 1.42; 100× oil immersion objective, NA 1.40) with FV10-ASW software or an Olympus FV3000 laser scanning confocal microscope (100× oil immersion lens, NA 1.49) with FV31S-SW software. Brightness and contrast were adjusted post-acquisition using either FV10-ASW or FV31S-SW software. Images in Fig. S1 were collected from three optical sections at a 0.35 µm step size, comprising a total of 0.7 µm depth. These images were then deconvolved using three iterations of the Olympus CellSens Deconvolution for Laser Scanning Confocal Advanced Maximum Likelihood algorithm. The images displayed in the figure show a single optical slice. Images in Fig. 1C and Movies 1 and 2 were collected as live time-lapse movies using the Olympus FV3000 resonant scan head. Three optical sections at a 0.36 µm step size (total depth 0.72 µm) were collected at a frame rate of 1.37 frames/s for Fig. 1C pre-biogenesis/Movie 1 and 1.40 frames/s for Fig. 1C post-biogenesis/Movie 2. For each sample, 250 frames were collected. Images were deconvolved as described above. The images in Fig. 1C represent a time maximum-intensity projection for a single optical slice; Movies 1 and 2 display a maximum-intensity projection of three optical slices played over time. For LysoTracker staining, LysoTracker Deep Red (Invitrogen) was diluted 1:1000 in PBS, and dissected salivary glands were incubated in this solution for 1 min on a shaker platform. Stained glands were rinsed for 1 min in PBS, then mounted and imaged immediately. Quantification of LAMP-GFP and Syt1-GFP tubule length and number was done using ImageJ. Quantification of *Sgs3-DsRED* granules containing markers (*Syt1-GFP*, *FYVE-GFP*, or *Rab7-EYFP*) was done in FV31S-SW software.

## Supplementary Material

Supplementary information
