# Supplementary material for: Mistargeting of secretory cargo in retromer-deficient cells
Source: Dis Model Mech. 2021 Jan 22;14(1):dmm046417. doi: 10.1242/dmm.046417 (PMC7847263; doi:10.1242/dmm.046417)
Supplement: Supplementary information [file dmm-14-046417-s1.pdf]

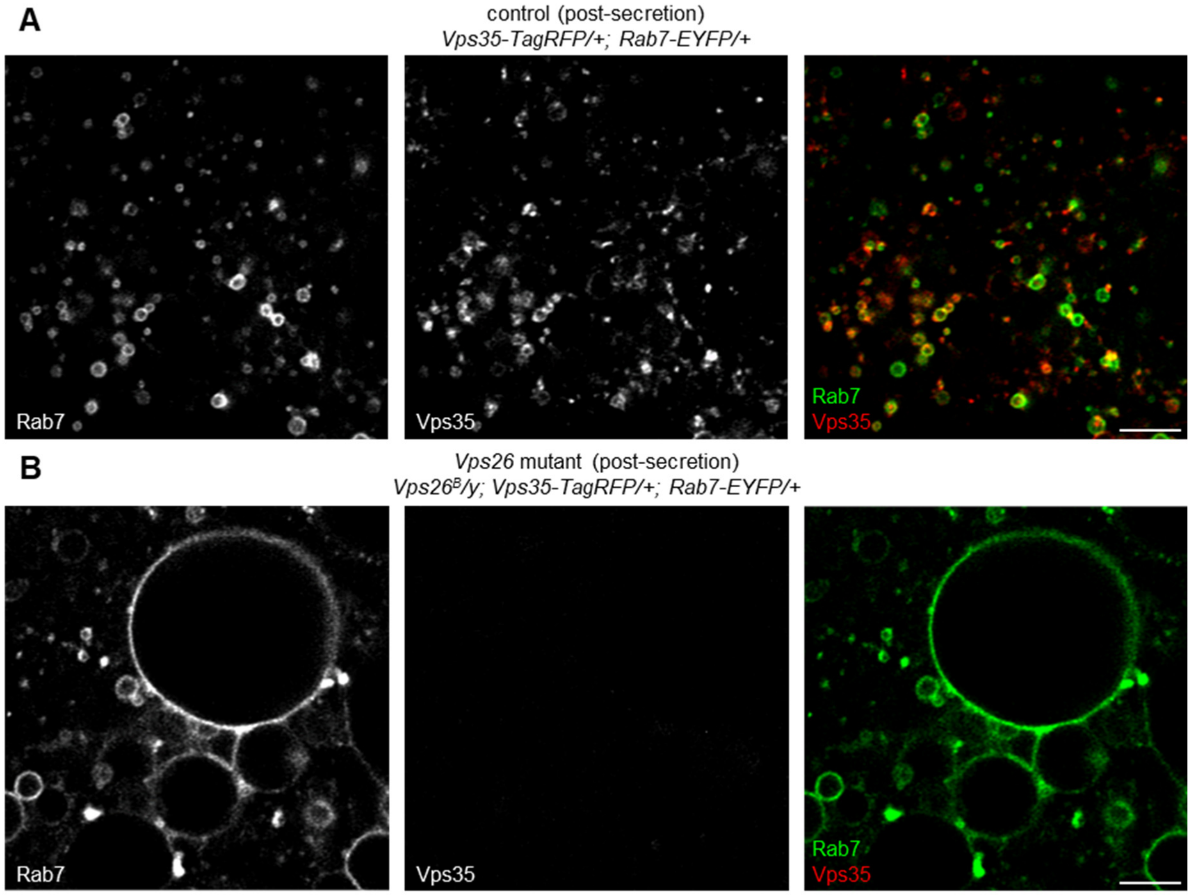

Figure S1. Vps35 protein is absent in *Vps26* mutant salivary gland cells. (A) Live-cell imaging of Rab7-EYFP (green) and Vps35-TagRFP (red) in control salivary gland cells post-secretion. As expected, Vps35 partially co-localizes with Rab7. (B) Live-cell imaging of Rab7 and Vps35 in *Vps26* mutant salivary gland cells post-secretion. Vps35 protein expression is undetectable, and Rab7-positive endosomes are significantly enlarged in *Vps26* mutant cells (see Fig. 4 and Fig. S2). Images were taken using identical laser power and acquisition settings. Scale bars 5 μm.

Disease Models & Mechanisms • Supplementary information

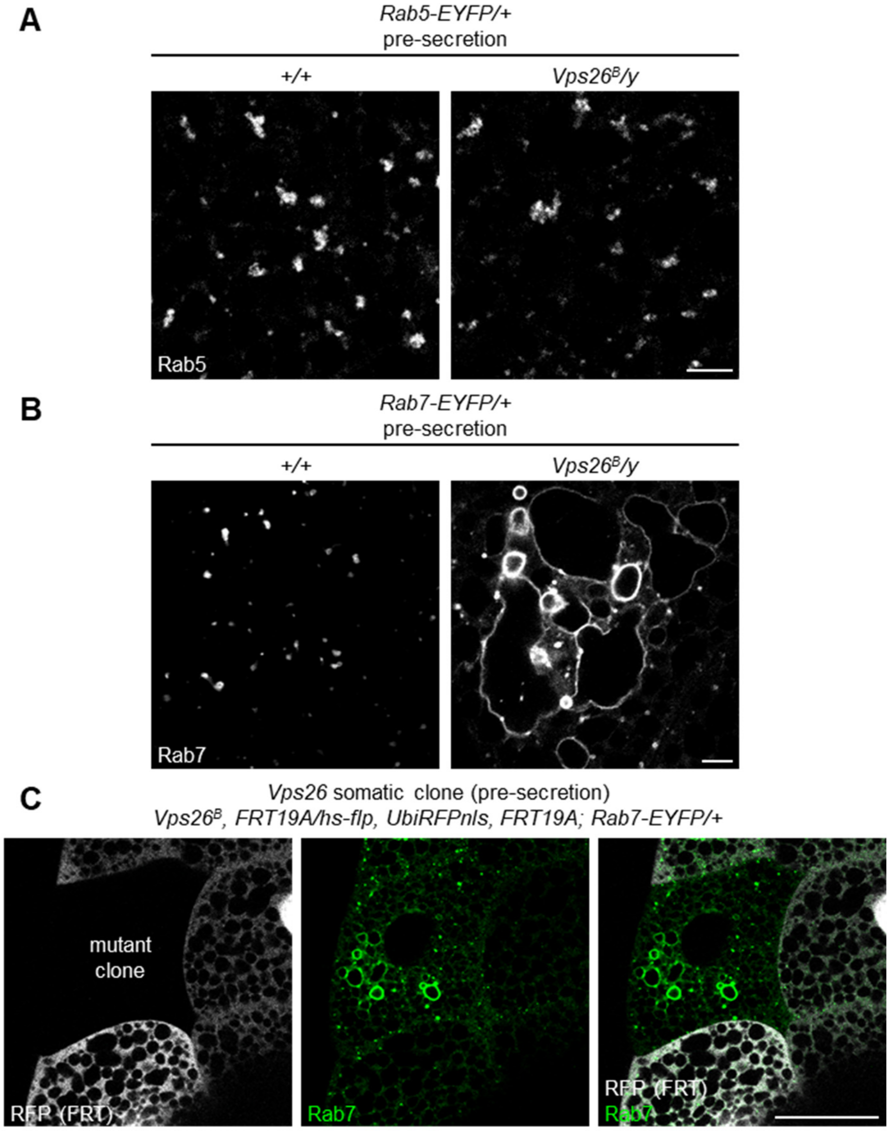

Figure S2. Analysis of endosomal morphology in *Vps26* mutant cells. (A) Live-cell imaging of Rab5-EYFP in control and *Vps26* mutant cells shows that the morphology of Rab5-positive endosomes appears to be unaffected in *Vps26* mutant cells. (B) Live-cell imaging of Rab7-EFYP in control and *Vps26* mutant cells shows that Rab7-positive endosomes are dramatically enlarged in *Vps26* mutant cells. (C) Somatic *Vps26* mutant clones show that Rab7 endosomal morphology defects are cell-autonomous. The mutant clone is marked by loss of RFP (shown in gray); Rab7 shown in green. Only the mutant clone contains enlarged Rab7-positive endosomes. Note that most endosomes are localized near the basal membrane in salivary gland cells, while the enlarged endosomes are found in the medial region of the cell. Scale bars in A, B are 5 μm; scale bar in C is 50 μm.

Disease Models & Mechanisms • Supplementary information

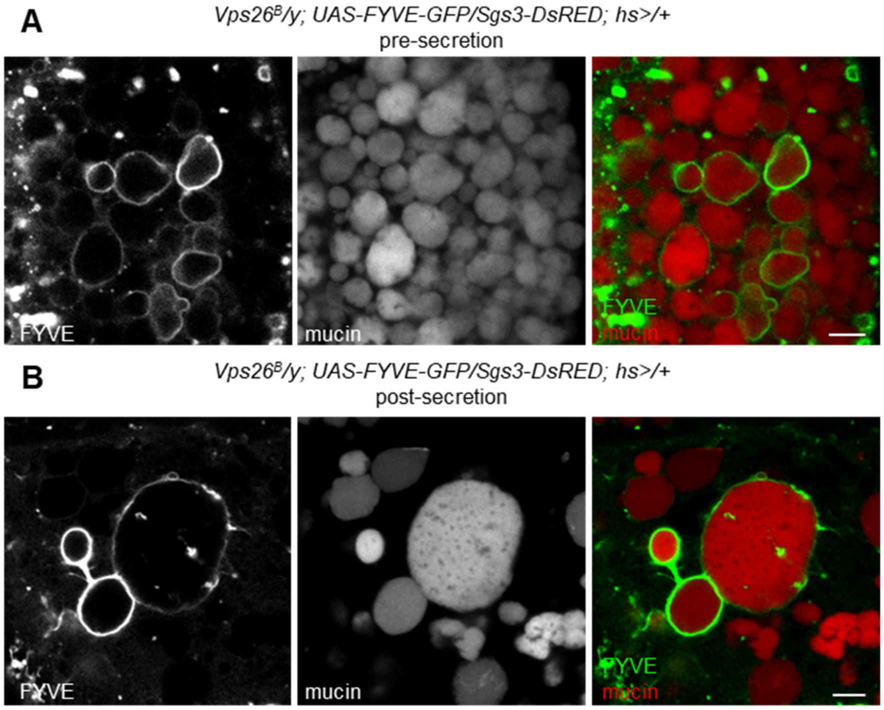

Figure S3. Enlarged mucin-containing vesicles are surrounded by FYVE-GFP in *Vps26* mutant cells. Live-cell imaging of the PI3P-binding marker FYVE-GFP (green) and mucins (Sgs3-DsRED; red) in *Vps26* mutant cells pre- (A) and post-secretion (B). At both developmental stages, enlarged mucin-containing vesicles are wrapped in FYVE-GFP. Scale bars 5 μm.

Disease Models & Mechanisms • Supplementary information

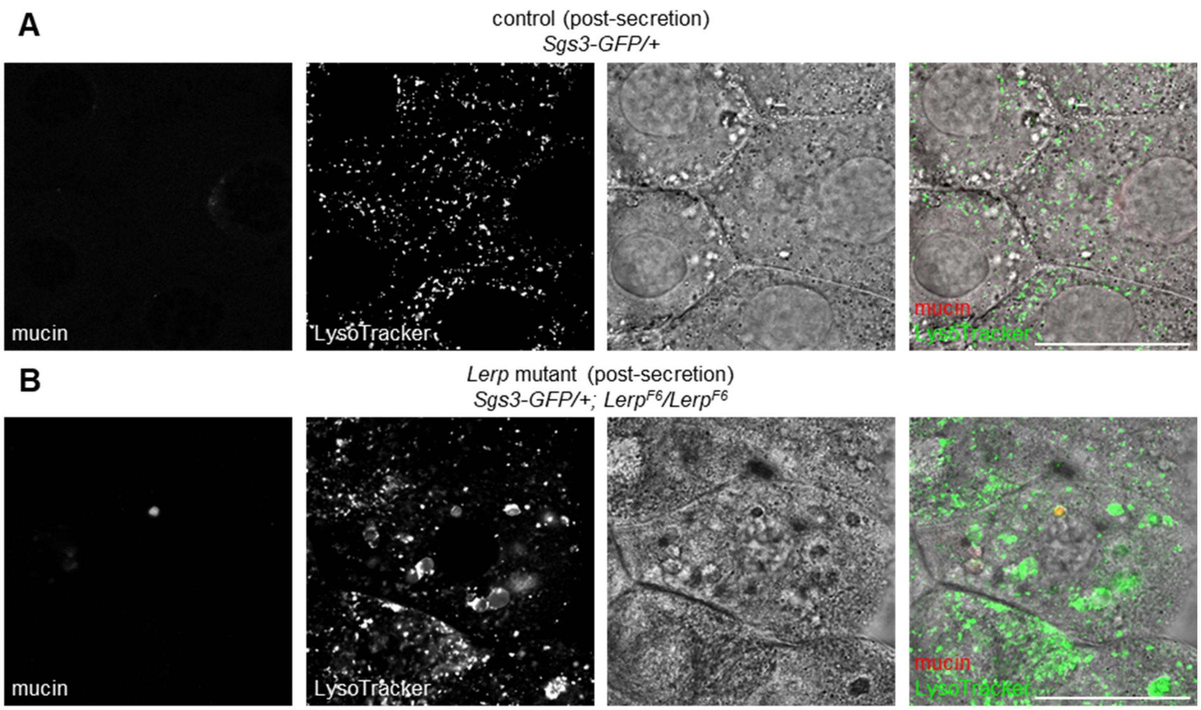

Figure S4. Lysosomal dysfunction does not cause mistargeting of secretory cargo proteins. (A) Live-cell imaging of mucins (Sgs3-GFP; red) and LysoTracker (green) in control cells post-secretion. All mucins have been secreted, and LysoTracker localizes in small puncta consistent with the size of acidified late endosomes and lysosomes. DIC image shows normal cellular morphology. (B) Live-cell imaging of mucins and LysoTracker in *Lerp* (fly CI-M6PR/lysosomal hydrolase carrier) mutant cells post-secretion. Mucins have been secreted, but many enlarged, LysoTracker-positive compartments are present. These enlarged, acidified compartments appear dark on a DIC image, suggesting the presence of undegraded material in dysfunctional, enlarged lysosomes and endosomes. Scale bars 50 µm.

Disease Models & Mechanisms • Supplementary information

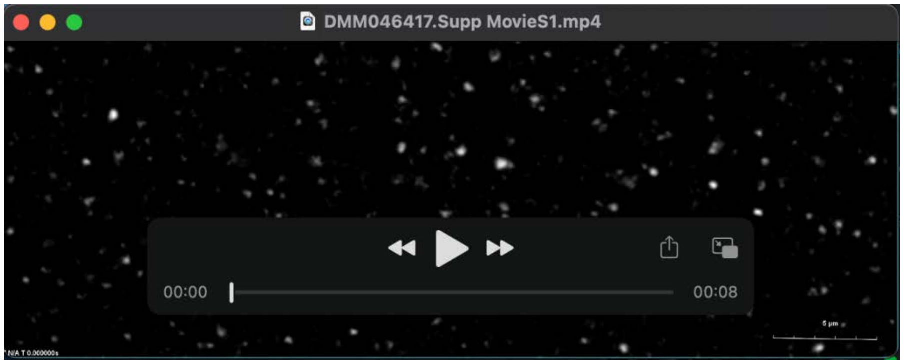

Movie 1. Time-lapse imaging of endosomal tubule activity in pre-biogenesis salivary glands. Live-cell time-lapse imaging of LAMP-GFP in salivary glands prior to the onset of glue biogenesis shows little tubule activity. Movie shows a maximum intensity projection of three optical slices; movie plays at a frame rate of 30 frames/sec. Scale bar 5 µm. A time-intensity projection of this movie is displayed in Fig. 1C.

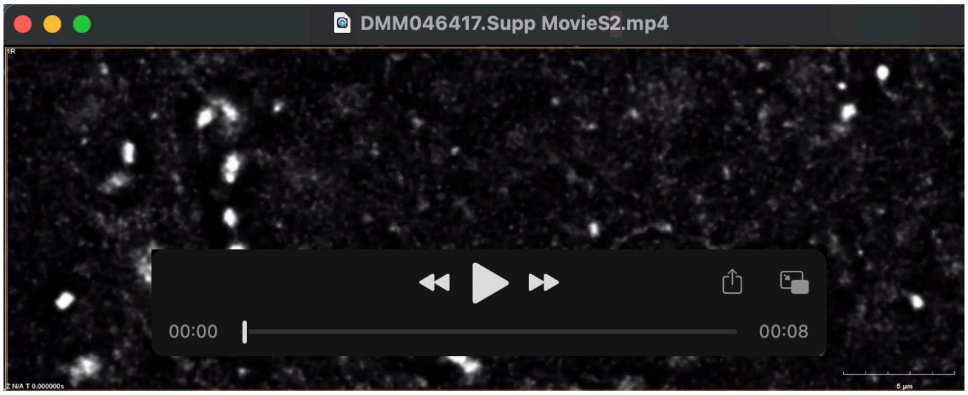

Movie 2. Time-lapse imaging of endosomal tubule activity in post-biogenesis salivary glands. Live-cell time-lapse imaging of LAMP-GFP in salivary glands after the onset of glue biogenesis shows the presence of dynamic endosomal tubules. Movie shows a maximum intensity projection of three optical slices; movie plays at a frame rate of 30 frames/sec. Scale bar 5 µm. A time-intensity projection of this movie is displayed in Fig. 1C.

Disease Models & Mechanisms • Supplementary information

Gene	Primer Sequence
Snx1 F	TTTGGCCTACAAGGTAACGAC
Snx1 R	ACTTGCCCACAAGCAGATCAT
Snx3 F	AAAGGAGTCCAGTGTCAGGC
Snx3 R	CTTCCATGCCTTTCCTGGGA
Snx6 F	GAGGAGTTCGTCTGGCTACAT
Snx6 R	AGGTGGACAGGGCGGAATAA
Vps26 F	GGTATCCGGCAAGGTGAACG
Vps26 R	TTACCCCGGTCGTAGTACAGT
Vps29 F	CGAGAACCTGACGTATCCGG
Vps29 R	AGGCCTCGAACTTGTACGTG
Vps35 F	ATGATGCCATCGACTTCGTG
Vps35 R	GCACCAGATTAGTGCCTACCA
Rab10 F	AGATGATCCTCGGCAACAAGT
Rab10 R	CCATAAACCGAATGCCATGTTCA

Table S1. Primer sequences for qPCR.

Disease Models & Mechanisms • Supplementary information

Figure Panel	Full Genotype
Figure 1C, 2D	*Sgs3-DsRED/UAS-LAMP-GFP; hs-GAL4/+*
Figure 2A, C-E	*Sgs3-DsRED/+; hs-GAL4, UAS-Syt1-GFP/+*
Figure 2B-D, F	*$Vps26^{B}$, neoFRT19A/y; Sgs3-DsRED/+; hs-GAL4, UAS-Syt1-GFP/+*
Figure 3A	*Sgs3-GFP/+* and *$Vps26^{B}$, neoFRT19A/y; Sgs3-GFP/+*
Figure 3B	*$Vps26^{B}$, neoFRT19A/hs-flp, UbiRFPnls, neoFRT19A; Sgs3-GFP/+*
Figure 3C, E	*Sgs3-DsRED/+; hs-GAL4, UAS-Syt1-GFP/+*
Figure 3D, F	*$Vps26^{B}$, neoFRT19A/y; Sgs3-DsRED/+; hs-GAL4, UAS-Syt1-GFP/+*
Figure 4A, C	*Sgs3-DsRED/+; Rab7-EYFP/+*
Figure 4B, D	*$Vps26^{B}$, neoFRT19A/y; Sgs3-DsRED/+; Rab7-EYFP/+*
Figure 5A, E	*Sgs3-DsRED/+; Rab7-EYFP/+*
Figure 5B, E	*$Vps26^{B}$, neoFRT19A/y; Sgs3-DsRED/+; Rab7-EYFP/+*
Figure 5C, E	*UAS-FYVE-GFP/Sgs3-DsRED; hs-GAL4/+*
Figure 5D, E	*$Vps26^{B}$, neoFRT19A/y; UAS-FYVE-GFP/Sgs3-DsRED; hs-GAL4/+*
Figure 6A, B	*UAS-APP-YFP/+; Sgs3-GAL4/+*
Figure 6C	*$Vps26^{B}$, neoFRT19A/y; UAS-APP-YFP/+; Sgs3-GAL4/+*
Figure S1A	*Vps35-TagRFP/+; Rab7-EYFP/+*
Figure S1B	*$Vps26^{B}$, neoFRT19A/y; Vps35-TagRFP/+; Rab7-EYFP/+*
Figure S2A	*Rab5-EFYP/+* and *$Vps26^{B}$, neoFRT19A/y; Rab5-EFYP/+*
Figure S2B	*Rab7-EFYP/+* and *$Vps26^{B}$, neoFRT19A/y; Rab7-EFYP/+*
Figure S2C	*$Vps26^{B}$, neoFRT19A/hs-flp, UbiRFPnls, neoFRT19A; Rab7-EFYP/+*
Figure S3A, B	*$Vps26^{B}$, neoFRT19A/y; UAS-FYVE-GFP/Sgs3-DsRED; hs-GAL4/+*
Figure S4A	*Sgs3-GFP/+*
Figure S4B	*Sgs3-GFP/+; $Lerp^{F6}$/$Lerp^{F6}$*

Table S2. List of full *Drosophila* genotypes.
